# Parent - adolescent communication on sexual and reproductive health: the qualitative evidences from parents and students of Addis Ababa, Ethiopia

**DOI:** 10.1186/s12978-020-00927-6

**Published:** 2020-05-27

**Authors:** Meseret Shiferaw Yibrehu, Bernard Mbwele

**Affiliations:** 1 School of Public Health, Gondar University, P.O Box 196, Gondar, Amhara Ethiopia; 2 RN, Midwife Nurse, Public Health Consultant, P.O. Box 30995, Addis Ababa, Ethiopia; 3 Department of Epidemiology and Biostatistics, University of Dares Salaam, Mbeya College of Health and Allied Sciences UDSM-MCHAS, PO Box 608, Mbeya, Tanzania; 4 Vijiji International, Mawenzi Road, P.O Box 7823, Moshi, Tanzania

**Keywords:** Parent-adolescent communication, Sexual reproductive health, Early pregnancy, Sexually transmitted infections, HIV/AIDS, Ethiopia, Addis Ababa

## Abstract

**Background:**

Repeatedly adolescents in Africa have been experiencing early pregnancy by more than 50%, early parenthood by 30% and new HIV infections by more than 80%. Parent - Adolescent communication as an effective strategy on sexual and reproductive health has not been taken up in most of African countries including Ethiopia. The aim of the study was to assess the challenges of Parent - Adolescent communication on sexual and reproductive health practices in Addis Ababa, Ethiopia.

**Methods:**

A qualitative cross-sectional study exploring Parent - Adolescent communication practices was conducted in two high schools Yeka sub-city, Addis Ababa, Ethiopia. Students aged 15–19 years were recruited for in-depth interviews and focused group discussions (FGD) as well as respective parents for in-depth interviews and parents’ FGD.

**Results:**

Twenty students were available for in-depth interviews and all of them for FDG. Sixteen parents were available for in-depth interviews and nine of them for parent’s FGD. Parent - Adolescent communication on sexual and reproductive was reported to be important by both adolescents and parents. The parental initiation is rare. The initial sexual activity by the adolescents triggers initiation by parents. The communications are gender dependent, not planned and not continuous and inhibited by intergenerational cultural taboo. A gap exists in parental knowledge on such communications. Parents deny responsibilities to communicate with adolescent as they fear it will perpetuate early sex practices, adolescents are too young it’s an embarrassment, often being busy for household income retards their wills to communicate.

**Conclusions:**

Parent-Adolescent communications on sexual and reproductive health is not a common practice in Addis Ababa, Ethiopia due to taboo, cultural structures, gender domains and parental knowledge. These findings alarm the risk of adolescent exposure towards unwanted pregnancies, transmissions of HIV/AIDS and Sexually Transmitted Infections in Addis Ababa.

## English plain summary

Adolescents in Africa have been at risk of more than 50% early pregnancy, 30% of early parenthood with more than 80% new HIV infections. Most of adolescents of Ethiopia are missing the parental guided communication and discussion regarding safe sex, contraception access and safe abortion if need. Continuously, adolescents are misguided by their peer groups, or siblings. As a result, most adolescents become prone to unsafe sex that leads to early pregnancy, HIV infections, and sexually transmitted infections. Communities of Ethiopia present undocumented explorations for challenges of Parent - Adolescent communication practices on sexual and reproductive health.

We conducted a qualitative cross sectional study in two schools of Yeka sub city, Addis Ababa, Ethiopia to assess the challenges of Parent - Adolescent communications on sexual and reproductive health practices in Ethiopia.

We found limited practices for Parent - Adolescent communications. Mostly communications are stimulated by adolescents’ initial attempts at sexual activity. The communications are gender dependent, not planned and not continuous. Intergenerational cultural and taboo limits the initiation of Parent - Adolescent communication. Some parents denies their responsibility to communicate with their adolescents as they fear it will perpetuate early sex practices and embarrassed by the process. Parental knowledge of sexual and reproductive health is reported to be low with parent’s perceptions that adolescents are too young for the communications often they are busy for household income.

The official guided parental training and raising of the awareness on Parent - Adolescent communication is missing. Community based campaigns targeting parent’s knowledge, attitudes and practices to initiate Parent - Adolescent communication are needed.

## Background

The World Health Organization (WHO) defines adolescents as those people between 10 and 19 years of age. It includes in the age-based definition of “child”, for a person under the age of 18 years where a specific health and developmental needs and rights are sensitive. It is also a time to develop knowledge and skills, learn to manage emotions, relationships, acquire attributes and abilities of assuming adult roles [1]. Our world is a home for 1.2 billion adolescents making up 16% of the world’s population [2]. This is the fastest growing population especially in the resource limited nations [3].

Due to difficulties accessing contraception and Adolescent Sexual and Reproductive Health (ASRH) interventions, there have been unsafe abortions ranging from 25 to 30% among adolescent pregnancies globally [4]. Repeatedly, adolescents in Africa have presented early pregnancy with more than 50% of their pregnancies. Un-intended early pregnancy is ranging from 18 to 29% amongst all pregnancies in all African countries [5]. It is estimated that 80% of new HIV infections are among adolescents with 4 times girls at risks than boys at risk globally [6, 7] often with limited knowledge [8]. Again the adolescents present with a highest incidence rate (256 per 1000) for Sexually Transmitted Infections (STI) i.e. Syphilis, Gonorrhea, Chlamydial infection and Trichomoniasis [9]. This population in Africa has reported to face challenges to persuade aspirations for their future life through education [10]. The main reason for the above scenario is the gap in promoting the Sexual and Reproductive Health (SRH) agenda focusing in African scenario [11].

Structural short comings of parenting adolescents has resulted to low uptake for the reproductive health services utilization among adolescent [12]. One of the short coming is the authoritarian and uni-directional, vague warnings from parents in Africa. Another one is gender power and cultural norms around sexuality [13], Evidence exists that most of parents in African countries do not take part in solving social and structural issues related to reproductive health [14]. As a result, unsafe abortions [15] and high maternal deaths appear to be common among Adolescents [4]. With this regard, effective communications between parents and adolescent is thought to be overlooked [16].

In efforts to improve adolescents’ reproductive health service utilization, Ethiopia has insisted the need for development of life-skills to facilitate Parent - Adolescent communications. One of the most developed areas is the initiation of adolescent centers [17].

Generally, communities of Ethiopia present low frequencies of communication on sexual and reproductive health issues [18] especially for parent and adolescent [19]. There is a notion that discussion about condom use will perpetuate more sexual acts by adolescents. According to the Ethiopian culture and taboo, parents feel ashamed. They also feel that they lack skills to initiate Parent - Adolescent communications on sexual matters [20]. Routinely, parents of Ethiopia have been focusing on the negative consequences of sexual intercourse in a non-friendly manner. As a result, most of the adolescent communication attempts regarding sexual matters is generally misguided by their peer group of the same sex [19]. Some literature from Ethiopia, mentions a lack of parent knowledge on SRH and low literacy being the main cause [13, 20]. While the dogma continues, adolescents have been at risk to sex at an early age (< 15 years) with an adverse exposure to Sexually Explicit Materials (SEM) without interventions [21]. Again, adolescents of Ethiopia are less likely to use condoms with unmarried, non-cohabiting adult partners due to high risk sexual behaviors [22] due to missing parental guidance from parents [22–24].

There is an evidence from published trials showing a need for effective parent-based interventions in improving communication [25, 26] however most of inventions were conducted in western countries [27]. For example in US, 11 trials indicated a medium effect on increasing communication, 9 trials had a large effect in parental comfort with communication and effects were positive regardless of delivery mode or intervention dose [28]. Mostly, these communications have a direct impact on reduction of HIV/STI [29] and unwanted pregnancies [30] following delayed sexual activity, condom use, and sexual communication among adolescents [27].

Although peer support for SRH and HIV/AIDS is useful [24], an open and respectful communication among parents and adolescents is more effective. The later construct provide a continuum of support of early sexual life among adolescents [31]. For the parents who missed this opportunity, their adolescents are more likely to seek information from peers and internet [21] which might misdirect them to further risky sexual behaviors [32].

There are many hypothesized risk factors for poor Parent - Adolescent communication. One of them is parental knowledge and others are cultural issues like embarrassment of parents [33]. Continuous research works providing evidence for state of art Parent - Adolescent communication practices are needed to determine when, how or on what incidences parents shall initiate the communications [34].

In Ethiopia, the documentation of factors affecting Parent - Adolescent communication as effective parental monitoring in combating unwanted pregnancies HIV, and STI [32] towards a safe SRH is missing [35]. Some quantitative information exists [33, 35, 36] but it lack detailed explorations [17]. The aim of this study was to assess the challenges of Parent - Adolescent communication practices on SRH and provide qualitative evidence to further guide new interventions.

## Methods

The study design was of cross-sectional study using the qualitative approach in providing reasons for limited Parent - Adolescent communication practices in families of Addis Ababa, Ethiopia.

The study was conducted in two high schools, namely of Wondirad public school and Hill Side private school of Yeka sub-city, Addis Ababa, Ethiopia. A total of 20 adolescent’s student’ aged 15–19 years were enrolled from two schools (ten each). Eligible students were those who were available to volunteer, willing to discuss sexual and reproductive health issues and had never attended any of ASRH programme. Parents of students with one or more adolescent children willing to be interviewed were respectively approached. The parents who were available and have time for interviews were approached.

From the group of twenty in-depth interviews for students, four groups of Focus Group discussions (FGD) were made from a mix of best contributors, moderate contributors and poor contributors of in-depth interviews. A total of four FGDs comprised of five to six students were conducted from each of the two schools separately. To consider gender balance in FGDs a ratio of 1:1 (male to female) ratio was used.

From respective twenty parents, all parents were asked to participate for interviews based on their willingness and availability by time. Questions for in-depth interviews covered existence of the communication, what cause them to initiate, most topics covered (puberty, safe sex, contraceptive methods, and abortion, STI or HIV) continuity and common challenges encountered of perceived. The parents were then approached for FGD to help eliciting different sources of challenges for Parent - Adolescent communications.

Focus group discussions were designed to offer detailed information and to gather insights gained by clarifications. The question guide for probing gathering of more information on the occurrence of Parent - Adolescent communication, topics discussed (puberty, safe sex, contraceptive methods, and abortion, STI or HIV) if communication was continuous and factors affecting the communications were collected once an attempt of communication was reported.

Collected demographic data and responses were gathered by open-ended questions that allowed probing occurrence of communications, topic discussed, timings, planning and challenges. The audio-taped interviews developed insights about how individual experiences, peer influences, and cultural norms interact to guide perspectives and individual behaviors.

All interviews were undertaken by a midwifery nurse who had an experience of working on ASRH. A research assistant, sociologist working for the programmes that help communities to solve young people’s social challenges, assisted the investigator in recording and note – taking. Discussions were made in Amharic language using digital recorders and stored into the investigator’s computer and the data management storage computer of School of Public Health, Gondar University with password protection.

### Data analysis

Data was transcribed from the audio tape into a word document narration word by word. It also was checked for completeness and consistency by cross checking it with the recorded tape. The transcribed data was translated into English language version. The transcriptions and translations were done sequentially, following data collection. The investigator had to read and re-read the transcribed and translated data so as to be familiarized with the contents. The investigator checked the connectivity of themes and concepts from both methods of data collection and reviewed substantive findings for causes of poor Parent - Adolescent communications that offer a meaningful implications of Parent - Adolescent communications in relation to ASRH agenda [31]. The transcript was then converted into plain text and exported to Open Code software. The exported transcript was then coded into different themes and then sub-coded into sub-themes. In reviewing the codes, careful limitation on not to make too much of the sub-coding was done. Codes were then aligned into important unifying concepts, and new findings into new existing codes that were found to fit using the Standards for Reporting Qualitative Research, (SRQR) [32]. The categorization the codes by collecting similar codes from in-depth interviews and FGD were done. Lastly, final themes were formulated by triangulation based on the pre-set themes within the study objectives.

### Ethical consideration

The study and its informed consent was approved by the University of Gondar and Addis continental institute of Public Health Institutional Review Board. Permissions to collect data from schools were granted by the School Principals following detailed explanation on the research objectives. School Principals gave consents as guardians that adolescents taking part in a research are protected by safety, rights and interests using the safeguarding principles of ESOMAR World Research Codes & Guidelines for Interviewing Children and Young People [37].

Invitations with appointment dates to eligible candidates for the interviews and FGD were given. The letter of introduction with a short explanation regarding the study objectives and expectations from the participants was given by investigator. The information sheet was attached with the letter, and showed the rights of the participants and confidentiality were addressed by using unique identification numbers and security of data was given. A request to the participant to state a contact address and suitable interview time if they are willing to participate in the study was made. A consent form that allowed participant to sign before participation was given. The entire interview was conducted individually and in private.

## Results

There were twenty adolescents from schools available for in-depth interviews and 16 parents available for in-depth interviews. There were a total of four FGDs for twenty students conducted and one FGD of nine parents with four parents were not available, six did not had time for discussions, one parent dropped out for FDG at the last minute. The following were the themes for Parent - Adolescent communication grouped as findings from Parents and Adolescent. Narratives are shown as explanations from in-depth interviews and comments or complaints from FGDs.

### Findings from students

#### Parent - adolescent communication on ASRH is important but missed

An adolescent boy sixteen years of age from one of the group discussion explained; “*It would have been good to have this type of discussion; I sometimes see these type of discussions on film, and wish if it could happen in my family*.”

#### Few parent-adolescent communication practices

An eighteen years old boy during in-depth interview explained “*After he told me about the changes during puberty the discussion stopped there. I had so many questions but I was afraid to talk to him. I was shy and afraid*” and he quoted his father’s words “*Is there anything to discuss after we discuss about puberty?”* A fifteen year old girl explained *“I tried once to ask about bleeding, but they both said I shall ask my aunt. This is how it works”.*

During the second FGD, three boys (Age and code names couldn’t not be tracked) loudly complained “*I prefer if they would have told us at early age, if they would have started the discussion earlier, we would have been open to discuss anything with them, but because they are not communicating about it we are doing everything secretly, and we share information with our friends and ignore them*”. Another 19 years of age added “*it is strange that they know we are doing sex but they don’t talk to us how it can be bad*”. The last one aged 18 years old commented “*if you initiate discussion they say you are not disciplined enough. Even when the television series is showing a sex relation scene. They switch off the television*”.

In the FGD, a 17 years old girl said; “*As I am part of the community my experience is not different from others, there is no discussion between me and my parents.*”

#### Cultural construct is the main issue

A seventeen years old boy explained; “*May be because it is not common in our Ethiopian culture, in our culture it is considered as an embarrassing to discuss about this issue*.” A seventeen years old adolescent girl also explained; “*It is the experience they have from their parents that distracts. There is no openness in what we do, it is a close relationship and no discussion between parents and children or they do not have this type of experience before.*”

Again a girl from one of the group discussion clarified; “*The first thing is culture, this is how they are brought up, they are brought up in a closed society*”.

#### Puberty time to initiate part of the communication

An eighteen years old boy explained during in-depth interview “*As he has gone through that process my father noticed my change and told me some changes I should expect through the process of adolescence*.”

An eighteen years boy from a group discussion commented; “*It would have been better if we discuss further because they will share us their experience and we will learn from them and improve things*.” A seventeen years old girl also discussed; “*It is not equal to face a problem after you are informed and before you are informed. I think many girls need this information before puberty*”. An eighteen years old girl commented; “*They do not give us proper advice on how to do it safely, instead, they threaten you and they show their power*”.

#### Gender and age influence on adolescent to communicate

A fifteen years old girl elaborated during interview; “*Yes, usually girls talk to their mothers, even boys prefer to talk to their fathers than their mothers. Unless the father is arrogant.*” An eighteen years old girl explained; “*But … because she is female, I usually discuss with my mother, in addition my mother is always available to have time and discuss*.”

Another boy, fifteen years of age, explained; “*I think it is easier to talk to our friends, whether it is about girlfriend or any change on our body it is easy to talk to our friends than our family. Because we are on the same age and generation we are free to talk to each other; but we are a different generation with our parents and they may think differently.*”

An adolescent boy aged sixteen from a group discussion commented; “*Not with my mother and father. It is easier with brothers and sisters, otherwise with mother and father it is not easy to discuss this issue in detail. Because we are almost similar in age with our brothers and sisters it is easier for us to discuss sensitive practice with them*”.

#### Parents deny their responsibility

A seventeen years old girl explained; “*I think the reason why they are not telling us is because they think we will get the information from school, especially from Biology classes.”*

#### The parents’ fear is the problem

An eighteen years old boy explained in the interviews; “*Our parents think giving the awareness on sexuality and reproductive health may push us to the wrong direction.*”

#### Parents feel to be embarrassed

A seventeen years old boy explained *“It was not easy; normally it is embarrassing for me to discuss sexuality and reproductive health with my parents.”*

#### Adolescent “know better”

An eighteen years old boy, commented during FDG, “*Most of them are not well informed about sexuality, reproductive health and family planning, there is no access and openness to discuss this type of exceptional issues; our parents have no awareness on this issue.”* Similarly a 17 years old boy clarified in FGD; “*In our community even if they want to be open there is lack of knowledge to talk about reproductive health openly.”*

#### Parents feel that adolescent are too young

Another girl 19 years of age FGDs elaborated. “*They always see us like little children; they do not accept our growth. They only wake up when something burst, but that time it is too late for everything*” An eighteen years old boy said “*The other thing is they consider me like a young or little child.*” Another 17 years old boy clarified in FGD “*We keep everything secret they do not think that we know such type of thing and they consider us like kids when we are at 12 grad.*”

#### Gender discrimination among boys and girls

A seventeen years boy complained in FGD, *“I think it is because we are boys, otherwise especially mothers will tell their daughters about change during puberty, but for boys parents do not think reproductive health information is relevant.”* Similarly a grade 15 years old boy commented “*They think that it has no value to discuss with boys, so it is not common to discuss sexuality and reproductive health issues with boys, as to my family there is only discussion with girls.*”

#### Parents are busy

A sixteen years old girl from one of FGD complained “*In the first place she has no time to sit and talk to me.”* A seventeen years old girl elaborated, *“Maybe because my father usually stays out of home for different activities he is busy.”*

### Findings from parents

#### Parent - adolescent communication on ASRH is important but missed

A mother of five boys and two girls who was 43 years old explained; “*We need to inform our children before they face the problem, after they face a problem the solution for the problem is different, so they need the information from us.*”

A sixty three years old father of seven mentioned; “*I think it is good to give time and discuss this issue with children.*” One father fifty-two years old, (four children) denied a need for the discussion on sexuality saying; “*My children are reserved to their reading and study, they have no time to be exposed to other things, I never thought that they will think about other things than their education, especially about opposite sex things.*”

#### Parent-adolescent communication has a limited practice

A forty-three years old father of three explained during interview; “*No there is no discussion, most of the time we didn’t consider it as a big issue and we never thought of discussing around this issue.*” In agreement with the above idea, a 43 years old father of four children also explained; “*I never discussed this issue with my children.*” Similarly a mother from a group discussion said “*How could you talk about this type of issue to your children? No one can talk about it.*”

#### Culture is the problem

A fifty-seven years old father (four children) explained; “*I think it is how I was brought up, and it is the influence of our culture”* This was also explained by a mother of three children, 42 years old; “*I think it is how I myself am brought up; my mother never discussed this type of issue with me. Similarly, I have no courage to sit down and talk about this issue with my children.*”

#### Selection of time to initiate

A forty-two years old mother of three children commented in FGD, “*When I see something different I will tell them to take care*”. A fifty-five years old father of three children commented; “*Most of the time we discuss about changes around adolescence and explain the reasons why.”*

#### Gender and age influence on adolescent to communicate

A father of five children aged 56 years explained; “*Fathers are there to threaten and give warnings otherwise I will refer the discussion to their mother and older sister and I will do the father’s part.*” A mother of six, 59 years old from a group discussion commented; “*Let them talk with their peers, even though we have no chance to hear what they are talking about*.”

#### Not their responsibility

A forty years old mother (three children), described her experience during interview “*She is learning it at school, so I never tried to talk about this issue with her.*” One of the mother aged 39 (five children) from the group discussion clarified; “*Today’s kids have lots of opportunities to gain knowledge on sexuality and reproductive health to mention some; school, TV, film and radio are the main.”*

#### The fear of parents on children’s early sexual engagement

A fifty-two year old father (seven children) explained; “*I thought if I discuss this type of thing with my children I may push them in the wrong direction.*”

#### Embarrassment of parents

A mother of sixty-two (five children) explained; *“I think I thought the issue is embarrassing, something you cannot say it”* This was again mentioned by another mother (forty-four, four children), a group discussants; “*It embarrasses me and my children to talk about it.”*

#### Adolescents know better

A mother of eight children, 62 years from FGD discussants said; “*When I try to tell them something they say we are the one who know about it, they say we have better knowledge about it than us.”*

#### Parents are busy

A forty three year old father (two children) commented in FGD; *“As to me when I go home I am usually busy with different things like reading I have no time to talk to my children”* In agreement with the above idea, a 55 years old mother said; “*We are struggling with so many things and we didn’t give them enough time.”*

## Discussion

Our study has provided a detailed qualitative evidences for limited practices and challenges for Parents - Adolescent communication on sexual and reproductive health to adolescents as requested by many previous quantitative studies [36, 38]. In this case we found most of adolescents wished to practice the communication on sexual and reproductive health more freely. Unfortunately there are more parental factors that limit the discussion.

We found the prominent parental factor linked to a strong cultural barrier that parents don’t talk about sex to their children. As a result, most of adolescent complained that parents do not talk to them. as similarly reported by Shilton and colleagues where they found that knowledge, Ethiopian national agenda, Ethiopian laws, resources, culture, and cooperation affected the utilization of World Health Organization’s preventing early pregnancy guidelines [39]. A quantitative study done by Binu and colleagues in Nekemte town Ethiopia, found inconvenient times, lack of privacy, religious rules, culture, and parent prohibition to be the main barrier for the utilization of reproductive health services [40]. Our study therefore has explained the tendency of adolescent to learn from friends, cousins, and the media [21] which increases risks of having early sexual activities with high rate of unwanted pregnancies and unsafe abortions [14, 24].

Adolescents are often aware that sex is a complicated act and they demand parental advice on sexuality when they start sexual practices as reported by Mekonen in North-Eastern Ethiopia [36]. We found that students adolescent are aware unwanted early pregnancies, STI, HIV/AIDS and unsafe abortions risks. However they are worried that majority of parental communication start after puberty or late after poor outcome as described in Oromia, Central Ethiopia [15].

Exceptionally, we found that adolescent were complaining for their parents to deny responsibilities. They mentioned that parents perceive their exposure for studying biology at school is enough. Often they complained that the age of 10–19 is fair for initial discussion but parents think it’s too early to talk about it. Often both boys and girls demand the communication but more emphasis has been laid to girls mostly. The adolescent of Ethiopia often need time to talk to parents but parent avoid such communications and explains to be busy.

There is a mix of experiences among parents. Majority of parent do not practice Parent – Adolescent communication due to cultural barriers. Similarly, social cultural practices and taboo were described by Browes and the Open University training modules have shown Ethiopian parents tend to consider such communications as not moral activities in raising children [39, 40]. A study in In Eastern Wollega, Ethiopia was also in-line with our finding [41]. However, in our case we found a growing awareness of the communication to be important despite inheritance of cultural taboos and norms that are wished to be transmitted to new generations. Those few who practice do not plan or monitor the outcome. Thus, parents have remained opportunistic for initiating and monitoring these communications as the case of Western Ethiopia [41].

Exceptionally, we noticed that some parents declared that there is a limited practice because they perceive such communications are not a big issue. Among those who reported the practice they presented higher parental age and parity.

Generally, parents expressed a dilemma on time to initiate such communications. Most parents declared to be reinforced by pubertal changes of their adolescents which is late and might require health care worker’s assistance as shown by Beckett in U.S.A [42]. Unfortunately, such intervention might not be practical in Ethiopia.

We found that parental knowledge and awareness to be a problem as reported in similar to studies for the impacts on knowledge [43]. The preference on the topics regarding sexual communications is important but missed in most of the attempts to communicate. A study in Northern Ethiopia reported the use of warnings by parents which is a barrier that further limit the communications [44].

We often found mothers are frequent communicators to daughters and fathers to boys. Mostly towards the end of adolescence. This gender determinant was similarly reported in a cross sectional study in Dire Dawa, Eastern Ethiopia [19]. It can be hypothesized that female adolescents are more affected by unwanted pregnancies than male adolescents. Again male adolescent are rarely participating in Parent - Adolescent communication on SRH. This phenomenon promotes risky sexual behavior for boys to continue approaching young girls for sex (most likely unprotected).

Surprisingly, some parents (especially fathers) think that it is not their responsibility but it’s something that can be done at school and or other relatives. They think if they initiate such a communication, it will facilitate adolescents to commit open and frequent risky sexual acts. They also fear that their children will be engaged to sexual activities earlier if they would have communicated earlier. This phenomenon leave a gap for HIV and STI risk in many parts of Africa [23].

We have learnt that parents tend to express their worries of being embarrassed when they are obliged to start talking about sex to their adolescent children as similarly as described in Uganda [45]. In Ethiopia, parents have shown a hard feelings to talk about condoms and safe sex as it is perceived as a lack of discipline. Concurrently, both parents and adolescents thought of knowledge of reproductive system as a subject in Biology taught in schools as a misguiding information. Parents think that the subject tells it all. However, adolescents think that something extra needs to be shared.

At last, interestingly parents mentioned their role to work for the family livelihood as barrier which was also reported by adolescents. On the other hand, adolescent presented it a complaint while parent reported being busy it as an excuse. This mounts a need for interventions to scale up Parent - Adolescent communications on SRH by targeting parents as described in United States [46]. This phenomenon is explained by the fact that un-planned and non-continuous communications have very little impact on SRH, STI and HIV/AIDS prevention [17, 47–50].

Again careful interventions for parents in modifying intergenerational cultural heritage described in Dire Dawa, Eastern Ethiopia [19] will benefit parents who deny responsibility, parents with to communicate, those who feel embarrassment, feelings that adolescents are too young, gender constrains, busy perception and general parental knowledge on sexual and reproductive health.

### Study limitations

Our study was part of Masters Research as a requirement for graduating for Public Health with a funding limitation that affected a catchment area in Addis Ababa Ethiopia.

Student enrolled in a study were from high schools with majority of them aged 15 to 19 years. The time and funding could not extend our data collection in primary school and ordinary secondary schools. The interviewer, was adult female midwife nurse from whom we assume there was a barrier in freedom of sexual health communication from adolescents, especially boys.

We couldn’t conduct in-depth interviews and Focus group discussions 20 parents instead we had only 16 parents for in-depth interviews and nine parents for FGD (6 mothers and 3 fathers). A total of four parents were not available and not reachable, six did not had time for discussions, one parent dropped out for FDG at the last minute. Some parents were not willing to be recorded during the interview, as a result we used written narratives of their responses. Other parents who agreed were not comfortable to discuss these issues in a group; as a result.

## Conclusion

Parent - Adolescent communications on sexual and reproductive health is not a common practice in Ethiopia mostly affected by taboos, cultural structures, gender domains as well as parental knowledge, on sexual and reproductive health. This is an obstacle to monitor the progress and the impact of communications in reducing unwanted pregnancies, STI and HIV/AIDS transmissions among adolescents. Parent - Adolescent communications need to be promoted in schools targeting adolescent to initiate open discussion with a special curriculum. Community based campaigns by hospital outreach programs and community health workers targeting parent’s knowledge, attitudes and practices to initiate Parent - Adolescent communication are needed.

## Data Availability

The datasets used and or analyzed during the current study are available from the corresponding author on reasonable request.

## Abbreviations

AIDS: Acquired Immune Deficiency Syndrome
ASRH: Adolescent Sexual and Reproductive Health
HIV: Human Immunodeficiency Syndrome
SEM: Sexually Explicit Materials
SRH: Sexual and Reproductive Health
STI: Sexually Transmitted Infection
